# Microbial mats as model to decipher climate change effect on microbial communities through a mesocosm study

**DOI:** 10.3389/fmicb.2023.1039658

**Published:** 2023-06-15

**Authors:** C. Mazière, R. Duran, C. Dupuy, C. Cravo-Laureau

**Affiliations:** ^1^Université de Pau et des Pays de l’Adour, E2S UPPA, CNRS, IPREM UMR 525—Bât. IBEAS, BP1155, Pau, France; ^2^La Rochelle Université, CNRS, UMR 7266 LIENSs (Littoral Environnement et Sociétés)—2, rue Olympe de Gouges, Bât. ILE, La Rochelle, France

**Keywords:** global change, ocean warming, ocean acidification, microbial mat, experimental ecology, mesocosms

## Abstract

Marine environments are expected to be one of the most affected ecosystems by climate change, notably with increasing ocean temperature and ocean acidification. In marine environments, microbial communities provide important ecosystem services ensuring biogeochemical cycles. They are threatened by the modification of environmental parameters induced by climate change that, in turn, affect their activities. Microbial mats, ensuring important ecosystem services in coastal areas, are well-organized communities of diverse microorganisms representing accurate microbial models. It is hypothesized that their microbial diversity and metabolic versatility will reveal various adaptation strategies in response to climate change. Thus, understanding how climate change affects microbial mats will provide valuable information on microbial behaviour and functioning in changed environment. Experimental ecology, based on mesocosm approaches, provides the opportunity to control physical-chemical parameters, as close as possible to those observed in the environment. The exposure of microbial mats to physical-chemical conditions mimicking the climate change predictions will help to decipher the modification of the microbial community structure and function in response to it. Here, we present how to expose microbial mats, following a mesocosm approach, to study the impact of climate change on microbial community.

## 1. Introduction

In the marine environment, climate change is predicted to have great and long-term impacts (Pachauri, 2014; IPCC, 2021). The increase in air temperature due to the enhanced greenhouse effect will impact first the surface water temperature and then the deep ocean temperature (Orr et al., 2005; Pachauri, 2014; Hutchins and Fu, 2017; IPCC, 2021). The global surface temperature of the ocean is projected to exceed 2°C (relative to 1850–1900 temperatures) for the 22nd century according to RCP8.5 (Pachauri, 2014). Moreover, global warming is predicted to release faster CO_2_ into the atmosphere, increasing its concentration from 390 ppm to 700 ppm by 2100 (Pachauri, 2014). This rise will lead to a decrease in pH between 0.3 and 0.4 units. Ocean acidification is associated with increasing concentrations of HCO_3_^–^, CO_2_, and H^+^ and decreasing concentrations of CO_3_^2–^. Thus, the global carbon cycle is directly affected by ocean acidification. Ocean models also predicted a decline in the dissolved oxygen inventory of 1–7% by 2100 (Schmidtko et al., 2017). These physical-chemical changes will have a strong impact on marine life. Microorganisms are abundant and diverse in the oceans, with 4 × 10^29^ cells in the deep oceanic subsurface, 5 × 10^28^ cells in the upper oceanic sediment and 1 × 10^29^ cells in the oceans (Flemming and Wuertz, 2019). They dominate the metabolic activity, for example, ensuring approximately half of the global primary production of the oceans (O’Brien et al., 2016). They play a central role in the biogeochemical cycles, as well as in the exchange of trace gases that have direct impacts on local climate (O’Brien et al., 2016). They are also crucial for the good functioning of the aquatic ecosystems being at the basis of food webs. Thus, marine microbial communities are expected to play a central role in the response of the ecosystem faced with environmental change because of their key functions in the oceans (Azam and Malfatti, 2007).

Microbial mats are laminated microbial structures (van Gemerden, 1993) observed at the water-sediment interface in several environments (Fourçans et al., 2004; Bolhuis et al., 2014; Mazière et al., 2021). They show a vertical stratification according to different physical-chemical gradients such as oxygen, sulphide, and pH. In such organization different interacting metabolisms coexist allowing microbial mats to be self-sustaining structure ensuring important ecosystem services in coastal areas (Pajares et al., 2015). In response to environmental changes, microbial mats can show various strategies due to their versatility and large diversity, as well as to their functional resilience (Bordenave et al., 2007). They represent ideal microbial community model to study the effect of global changes.

Current climate change effects on microbial communities can be investigated but it is difficult to simulate the conditions induced by climate change *in situ*, especially the acidification that remains difficult to control in open natural environments. Different approaches have been developed to study climate change effects on organisms inhabiting marine environments. Some studies focused on specific natural environments defined as models where one or several environmental parameters are similar to those predicted by IPCC, such as hot springs to simulate a high water temperature (Kitidis et al., 2011; Danovaro et al., 2017). However, the microorganisms inhabiting these environments are already adapted to the physical-chemical changes occurring on them and no temporal changes comparable to those due to climate change are observed. Moreover, the environmental complexity does not allow a definitive conclusion on the cause(s) of the observed changes. Thus, the precise impact on the environmental functioning and the dynamic of the microbial community in response to climate change cannot be studied.

Global changes are rapid simultaneous changes of environmental factors due to anthropogenic activities which threaten marine microorganisms. So far, little knowledge has been acquired to predict the effect of combined alteration of several environmental factors on marine microbial communities. Understanding the microbial communities shifts in response to the modification of environmental factors would allow to predict the effect of global changes on ecosystem functioning (Pajares et al., 2015). In order to gain such knowledge, it is thus necessary to develop accurate and controlled strategies to expose model microbial community to controlled climate change conditions. The mesocosms are a good approach because they allow the implementation of artificial scenarios by controlling physical-chemical parameters mimicking as close as possible the natural environment or the environmental conditions to be simulated (Cravo-Laureau and Duran, 2014). It is also possible to distinguish each factor independently allowing thus the characterization of their precise impact. Other advantages are that replicates and analysis can be multiplied, as well as manipulations can be performed without the constraints encountered *in situ* (Petersen et al., 2009). Data collection and analysis are thus facilitated. Mesocosms are enclosed ecosystem experiments that have gained in popularity as research tools in ecological science, particularly in the study of coastal aquatic environments (Petersen et al., 2009). Mesocosms have been developed for many applications, including the study of environmental stress (Pajares et al., 2012), hydrocarbon degradation (Cravo-Laureau and Duran, 2014), simulated oil spill (Chronopoulou et al., 2013), climate change simulation (Stewart et al., 2013; Mazière et al., 2022). Mesocosms have been used to analyse the behaviour of specific microbial communities (Chronopoulou et al., 2013; Stauffert et al., 2013), and of complex microbial structure such as biofilm (Agogué et al., 2014) and microbial mats (Mazière et al., 2022). They allow the control of different physical-chemical parameters and the association of them that can act synergistically (Baragi et al., 2015; Baragi and Anil, 2016; Li et al., 2016; Mazière et al., 2022), which thus allows to disentangle the impact of the studied factors from other environmental factors (Cravo-Laureau and Duran, 2014). So far, most studies have focused on the impact on one specific population or microorganisms (Widdicombe et al., 2009; Beman et al., 2011; Gingold et al., 2013; Guanyong et al., 2017; Lee et al., 2017; Tan et al., 2019) in a specific environmental compartment, such as water column and deep-sea benthos (Danovaro et al., 2017). However, the investigation of complex microbial assemblages is necessary to obtain data representative of the reality (Dimitriou et al., 2017). For that, a good microbial community model, representing a complex network of microorganisms, diversified at both the taxonomic and functional levels, is required. Microbial mats have been shown to represent good microbial community models in experimental ecology, particularly revealing the effect of oil spills on microbial community structure (Bordenave et al., 2004b,^2007^) and functioning (Bordenave et al., 2004a,^2008^). Moreover, microorganisms inhabiting microbial mats are well-adapted to physical-chemical fluctuations (Fourçans et al., 2006) making them adequate models to study climate change in microbial ecosystems. To the best of our knowledge, only a few studies have reported the impact of climate change on microbial mats (Verleyen et al., 2010; Mazière et al., 2022). In this paper, an experimental methodology is proposed where microbial mats were used as a microbial community model in a mesocosm experiment, representative of coastal areas, which showed to be a good way for deciphering climate change effect on complex microbial community.

## 2. Materials and methods

Climate change was simulated by ocean acidification and warming according to the most pessimistic scenario (RCP8.5) predicted by IPCC (Pachauri, 2014).

### 2.1. Mesocosms design

Microbial mats for mesocosm experiments came from a non-exploited salt marsh located in Ars-en-Ré (46°13′29.9″N 1°31′07.5″W, Ré Island, France). These microbial mats were selected as microbial model to study the climate change impact because they presented layers more developed than those from exploited ponds (Mazière et al., 2021). A constant water height of 3 cm was maintained above them by the salt marsh owner to prevent microbial mats from being exposed to the air. Microbial mats were sampled with a core collector (2 cm mat and sediment depth), and transferred while maintaining their integrity, into mesocosms boxes (48 × 33 cm). Their transport to the laboratory was done without water, in the dark and at room temperature to avoid the destruction of the vertical structure.

**FIGURE 1:**
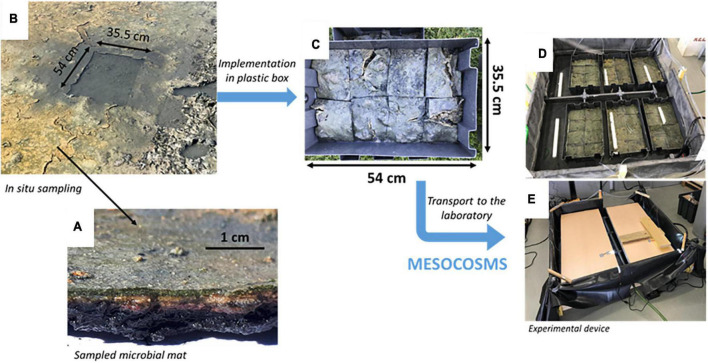
*In situ* sampling of the microbial mats for mesocosms experiment. Microbial mats were placed in containers with the sediment samples and transported to the laboratory for the mesocosms experiment. **(A)** Sampled microbial mat and its laminated structure. **(B)** *In situ* sampling of the microbial mat with a mold. **(C)** Implementation of the sampled microbial mat cut in eight parts to transfer it by avoiding damaging it as much as possible in a representing one mesocosm. **(D)** Six mesocosms circled by a pool representing one treatment. **(E)** The same than panel **(D)** with the light system above.

Four sets of six mesocosms were built, each representing a treatment (Figure 1). The water was distributed independently from the seawater reservoir on each microbial mats *via* stainless steel taps. This seawater was filtered at 80 μm and passed under UV light to prevent the presence of other marine organisms in the mesocosms system. The salinity was adjusted to that measured *in situ* [60 practical salinity unit (psu)] by adding salt coming from the salt marshes of the Ré Island.

A stable water level was maintained at 3 cm above the mat with a water outlet in front of its inlet. The microbial mats in salt marshes were under a continuous flow-through water depending on the evaporation rate and thus, of the daily temperature making the determination of the water flux difficult. The microbial mats in the mesocosms were under a continuous slow (0.5 L/h; dilution rate estimated around 10^–5^/h; water turnover in 1h20) flow-through water diffusion simulating a slow water inlet occurring in salt marshes, which ensures water renewal supplying natural nutrients for the development of microbial mats (Figure 1). The microbial mats were illuminated by LED (TOP-24H company by SYLED, France) 12 h a day following the day/night cycle observed during the spring in France (Figure 1). They provided white cold light colour, limiting the evaporation rate, and intensity of 12 ± 1 μmol.photons.m^–2^.s^–1^ (corresponding to 874 ± 70 lux) (HOBO Pendant^®^ Temperature/Light Data Logger, Onset Computer Corporation, USA) on the surface of the microbial mats. The LED approximates a solar spectrum according to the manufacturer’s instructions. It is certified like a natural light, used for the cultivation of aquatic plants.

**FIGURE 2:**
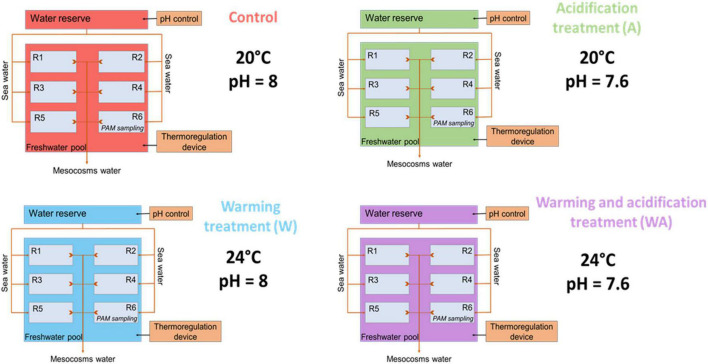
Schematic representation of the experimental design with the control (C), acidification (A), warming (W), and mixed (WA) treatments. The letter R represents the replicates. The first five replicates were used for sampling to perform the analysis listed in Table 1 and the sixth was dedicated to evaluate the photosynthetic activity potential measuring the chlorophyll *a* fluorescence by pulse amplitude modulation (PAM) and physical-chemical gradients. This figure was modified from Mazière (2021) and Mazière et al. (2022).

For each treatment, the desired temperature water above the microbial mats was maintained by placing the six containers with the sediment samples in a thermostated water bath with a pump (EHEIM universal 600, Germany) connected to a thermoregulating device (Teco^®^) (Figure 2).

### 2.2. Simulated scenarios

In order to acclimate the microbial mats to their new environment, a stabilisation period was applied (Stauffert et al., 2013; Gette-Bouvarot et al., 2015) maintaining the temperature (20°C) and salinity (60 psu) observed *in situ*. In this experiment, this period lasted 5 weeks ending when the physical-chemical parameters (salinity, temperature, pH, and dissolved oxygen concentration) in the water above microbial mats were stabilised for 1 week [see figure 2 in Mazière et al. (2022) and figure A in its Supplementary Materials].

A control (C) in which the parameters were not changed, and three treatments were then applied to the microbial mats for seven additional weeks [see figure A in the Supplementary Materials of Mazière et al. (2022)]. The first treatment was the warming treatment (W) in which the temperature was increased by 0.5°C every 2 days for 2 weeks until reaching 24°C. The acidification treatment (A) represented the second condition. Water acidification was performed in the water reservoir with the addition of CO_2_ (IKS aquastar, iks ComputerSysteme GmbH, Germany), allowing a drop of initial water pH of 0.1 unit every 4 days for 2 weeks until reaching a decrease of the initial pH of the water reservoir equal to 0.4 unit, i.e., 7.6. The water pH in the water reservoir was precisely monitored with a pH probe (826 pH mobile, Ω Metrohm, Swiss) to adjust the pH on IKS device by taking into account the alkalinity using the seacarb package on RStudio software (version 4.2.1© RStudio, R Core Team, 2020). This probe was calibrated in standard solutions (pH 4, 7, and 10, HANNA (instruments, France). The third treatment combined warming and acidification treatments (WA). Acidification and water warming were performed over 2 weeks to mimic a continuous temporal variation and not a sudden change in these parameters like the predicted IPCC scenarios (Pachauri, 2014). The mesocosms were then maintained with the stable conditions for five further weeks.

Many parameters were monitored to allow the most accurate experimental ecology approach possible, combining physical-chemical, diversity, functional, and biochemical analyses (Table 1). The sampling strategy was described by Mazière et al. (2022). Briefly, measurement of all these parameters was done before the change of conditions (t9) and regularly during their variation and stabilization (t16, t23, t30, t37, t44, t51, and t58). Among the six replicates, one was specifically dedicated for the evaluation of the community’s maximum potential for photosynthetic activity by measuring the chlorophyll *a* fluorescence with pulse amplitude modulation (PAM) as described in Mazière et al. (2022). The specific replicate was dedicated to PAM analysis because sampling for the analyses listed in Table 1 were performed immediately beforehand, leading to a suspension of biological material, which can disturb PAM analysis.

**TABLE 1:** List of samplings performed from the mesocosms for the different analyses.

Sampled material	Analysis		References
Microbial mat	Diversity		Mazière et al., 2021
Microbial mat	Cell number		Lavergne et al., 2014
Microbial mat	EPS		Mazière et al., 2022
Surface water	EPS		Mazière et al., 2022
Microbial mat	Pigment composition		Mazière et al., 2022
Microbial mat	Meiofauna diversity and abundance		Mazière et al., 2021
Microbial mat	Bacterial production		Garet and Moriarty (1996) modified by Pascal et al. (2009)
Surface water	Bacterial production		
Surface water	Nutrients	Nitrate, nitrite, ammonium, phosphate	Aminot and Kérouel, 2007
		Silicon	

A total of eight sampling points were done after the stabilisation period of 5 weeks. EPS, extracellular polymeric substances.

### 2.3. DNA extraction and sequencing

Microbial mats subsamples of 0.25 g were done and used for DNA extraction using the DNeasy PowerSoil kit (Qiagen) according to the manufacturer’s instructions. The bacterial V3-V4 region of the 16S rRNA gene was amplified using the primers 344F (5′-ACGGRAGGCAGCAG-3′) and 801R_m (5′-ACCAGGGTATCTAATCCT-3′) (Liu et al., 2007). PCR mix consisted in 12.5 μL of AmpliTaq Gold 360 master mix (Applied Biosystems), 1 μL of each primer (10 μM) and 1 μL of genomic DNA, in a final volume of 25 μL (adjusted with distilled water). All amplifications were performed on a Veriti 96 Well Thermal Cycler (Applied Biosystem) using the following PCR program: 10 min at 95°C, 30 cycles of 30 s at 95°C, 30 s at 63°C, and 40 s at 72°C, and finally, 10 min at 72°C. The sequencing was performed by the Genomic platform of Roscoff (France), using Illumina MiSeq technology.

### 2.4. Statistical analyses

The bioinformatic analyses on the DNA raw data obtained after sequencing were performed using the SAMBA (Standardized and Automated MetaBarcoding Analyses workflow) (v3.0.0) workflow written by SeBiMER, the IFREMER’s Bioinformatics Core Facility (Noël et al., 2021). Two replicates of the control condition at t44 and t58 were removed because they didn’t pass the quality control test during the data integrity step of the workflow. The taxonomic affiliation was performed against the Silva database v138 (Quast et al., 2012; Yilmaz et al., 2013) with 99% of similarities. All calculations and statistical analyses were performed on RStudio software (version 4.2.1 (RStudio, R Core Team, 2020). The statistical analyses used to interpret the physical-chemical data, the pigmentary composition, and the extracellular polymeric substances (EPS) concentration were described in Mazière et al. (2022). Non-metric multi-dimensional scaling (NMDS) analysis was based on the Bray–Curtis distance matrices calculated from the bacterial Amplicon Sequence Variants (ASVs) table (Supplementary Table 1). In order to define whether the treatment at a sampling time explained the variance among the Bray–Curtis distance matrices, a permutational multivariate analysis of variance (PERMANOVA) was performed with the *adonis* function of the vegan package. Linear discriminant analysis effect size (LEfSe) (Segata et al., 2011) on the 1,000 more abundant ASVs was performed on Galaxy web application to determine bacterial genera biomarkers for each treatment. The non-parametric Kruskal–Wallis sum-rank test (α = 0.05) was performed to detect taxa with significant differential abundance. The biological consistency was investigated by performing a pairwise Wilcoxon test (α = 0.05). A linear discriminant analysis (LDA) threshold score of 2.0 was applied.

## 3. Results and discussion

Although the decrease of 0.4 unit of pH (upH) in the seawater reservoir of the acidification treatments was effective, the water pH was not significantly different between the treatments [see figure 2 in Mazière et al. (2022) and figures B, D in its Supplementary Materials]. Such phenomenon has been previously described; it can be explained by abiotic factors controlling the CO_2_ chemistry in seawater (Zeebe and Wolf-Gladrow, 2001) and by biotic factors linked to oxygenic photosynthesis (Giordano et al., 2005; Crawfurd et al., 2011; Ma et al., 2019). Thus, it can be hypothesized that the acidification might result in CaCO_3_ dissolution, which in turn consume the protons in excess counteracting the acidification (Middelburg et al., 2020), explaining that pH was not decreased in our experiment. Regarding the biotic factors, it is known that many microbial species possess mechanisms allowing the storage of high CO_2_ concentrations named carbon concentration mechanisms (CCMs). The CCMs have been described in oxygenic phototrophs like algae (Giordano et al., 2005) and cyanobacteria (Ma et al., 2019). The storage of the CO_2_ added for acidification in CCMs might also explain why the decrease of pH was not observed in our experiment. However, when the maximum CO_2_ concentration capacity of the CCMs will be reached in the cells, it could be expected to observe a pH decrease in the water column due to the added CO_2_ (Black et al., 2019). Moreover, the dissolved oxygen concentration was observed to be more increased than in the other treatments, likely due to an increase in photosynthesis because of the carbon input (Black et al., 2019; Mazière et al., 2022). As expected, the temperature increased by 4°C from the initial temperature in the warming treatments [see figure 2 in Mazière et al. (2022)]. An increase in salinity was also observed in the warming treatments probably because of water evaporation [see figure 2 in Mazière et al. (2022)].

Thus, the mesocosms experiment allowed to change and maintain the conditions as expected (acidification of the water input and water warming on the mesocosms) (Petersen et al., 2009; Cravo-Laureau and Duran, 2014; Mazière et al., 2022). Such changes in environmental conditions are difficult to produce *in situ* because of the environmental complexity (day/night alternation, weather hazards, human impact…). By changing the conditions one by one, the changes in the other measured parameters could be attributed to the treatment. In our study, the warming treatment impacted the salinity whereas the acidification treatment affected the dissolved oxygen concentration. Thus, the mesocosms were effective to control the physical-chemical parameters for simulating climate change.

**FIGURE 3:**
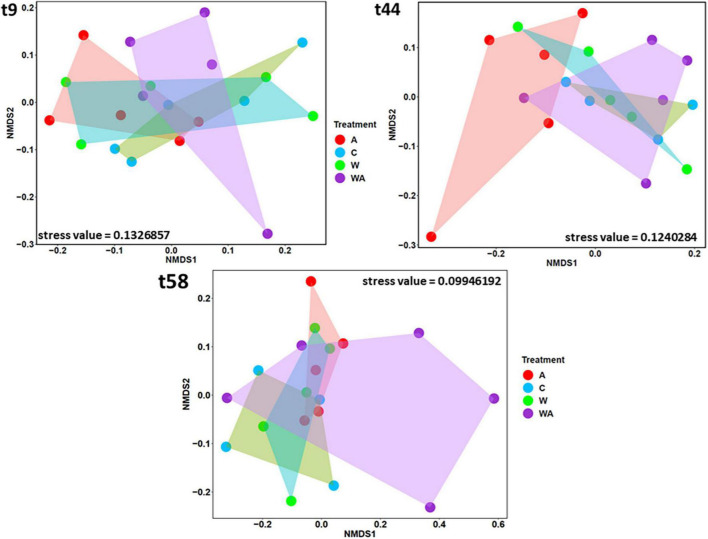
Comparison of bacterial communities according to the treatments. The Non-metric Multi-dimensional Scaling (NMDS), based on the bacterial 16S rRNA gene sequences, was performed to compare the bacterial communities according to the treatments [control (C), acidification (A), warming (W), and warming and acidification together (WA)] at the beginning (t9), the middle (t44), and the end (t58) of the experiment. The shapes correspond to the replicates (*n* = 5, except for control *n* = 4 at t44 and t58).

After changing the conditions, the bacterial community composition differed in the acidification treatment 3 weeks after (t44) in comparison to the other treatments (Figure 3) (PERMANOVA, *p* < 0.05). At the end of the experiment (t58), no more difference was observed between the acidification treatment and the control and warming treatments (Figure 3), suggesting that the bacterial community was resilient. However, the bacterial community composition differed, certainly due to the WA treatment (PERMANOVA, *p* < 0.05).

**FIGURE 4:**
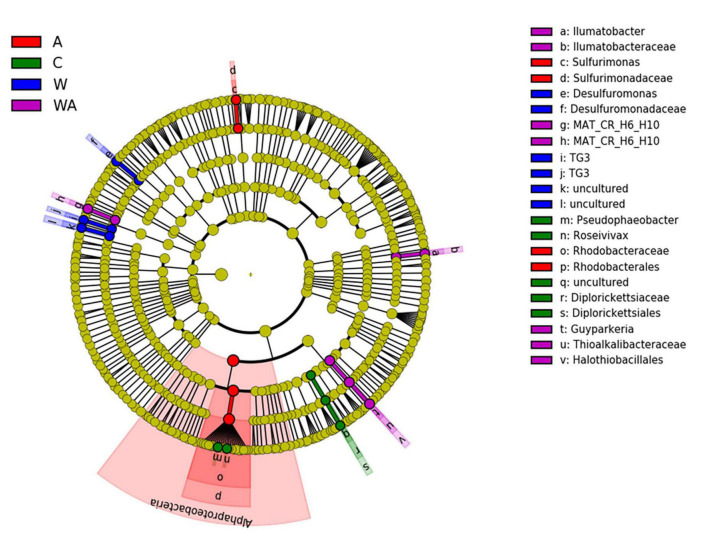
Linear discriminant analysis effect (LefSe). Comparison of bacterial communities at t58 in the different treatments [control (C), acidification (A), warming (W), and warming and acidification together (WA)] by linear discriminant analysis effect size (LEfSe). The analysis was performed with the 1,000 more abundant bacterial ASVs of each treatment.

Indeed, LEfSe analyses on the 1,000 more abundant ASVs revealed bacterial taxa differentially abundant between the four treatments at t58. LEfSe revealed that the acidification was characterized by bacterial biomarkers, genera significantly more abundant in the acidification treatment, belonging to genera affiliated to Rhodobacteraceae and *Sulfurimonas* (Figure 4). The *Sulfurimonas* species are widespread in the environment and present large flexibility to colonize various habitats due to their versatile energy metabolisms and their adaptive abilities (Han and Perner, 2015). They play key roles in sulphur, hydrogen, nitrogen, oxygen, and carbon cycles (Han and Perner, 2015). The *Sulfurimonas* species are known to grow over a large range of pH (Inagaki, 2003; Takai et al., 2006) with the capacity to tolerate oxygen (Sievert et al., 2008), features allowing *Sulfurimonas* to outcompete under the conditions observed after acidification in our experiment. The Rhodobacteraceae play a key role in biogeochemical cycles and are often eukaryote mutualists (Simon et al., 2017). The abundance of Rhodobacteraceae has ever been shown to be affected under a high *p*CO_2_ (Hu et al., 2021). *Desulfuromonas* genus, sulphate reducer bacteria (SRB) obligate anaerobe and obligate sulphur reducer (Margulis and Chapman, 2009), was revealed as a bacterial biomarker in the warming treatment (Figure 4). SRB are known to exhibit versatile metabolism, an asset to adapt to the modification of environmental conditions in various ecosystems (Giloteaux et al., 2013), particularly in microbial mats (Fourçans et al., 2008). The WA treatment presented *Guyparkeria*, *Ilumatobacter*, and MAT-CR-H6-H10 as bacterial biomarkers (Figure 4). *Guyparkeria* genus includes autotrophic sulphur oxidizers (Nosalova et al., 2022). *Ilumatobacter* species are generally found in contaminated places by hydrocarbons (Peng et al., 2015; Papale et al., 2020). MAT-CR-H6-H10 was described in a hypersaline microbial mat (Kirk Harris et al., 2013). The results suggest that the different treatments simulating climate change impact bacteria playing a role in the sulfur cycle. The acidification treatments (A and WA) affected sulfur oxidizers while the warming treatment impacted sulphate reducers.

Concomitantly, the increasing of the concentration of bound carbohydrate EPS in the acidification treatment was observed (Mazière et al., 2022) indicating that EPS are involved in the adaptation of communities to their environment as previously proposed (Dupraz and Visscher, 2005; Hubas, 2018; Prieto-Barajas et al., 2018). The most probable explanation is that the studied microbial mats are already naturally exposed to fluctuating environmental conditions (pH, light, temperature, salinity, etc.) with a great amplitude in salt marshes (Fourçans et al., 2004, 2006, 2008; Abed et al., 2007; Boujelben et al., 2012; Baumann et al., 2015; Mazière et al., 2021). Therefore, they are already confronted to temperature such those predicted by the IPCC for 2100. Changes in the bacterial metabolism probably enabled bacteria to maintain a high activity potential when exposed to the conditions of the simulated scenarios. Noteworthy, a change in the pigment composition of the microbial mats submitted to acidification was observed (Mazière et al., 2022), that suggested a shift in the photosynthetic capacities corresponding to a modification in the photosynthetic communities or their metabolism (Mazière et al., 2022). The microbial mats maintained in mesocosms allowed us to observe the effect of the acidification alone on photosynthetic microorganisms, which was not observable in the treatment combining both acidification and warming.

**FIGURE 5:**
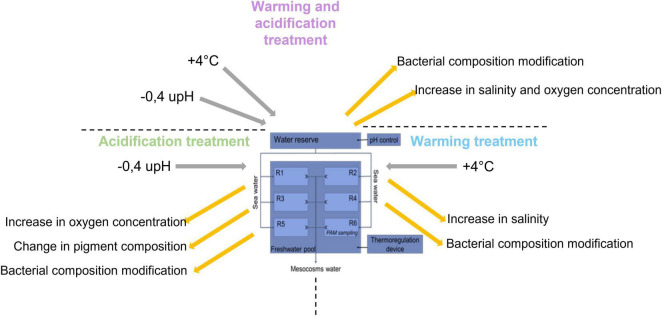
Summary of the impact of climate change on microbial mats.

To summarize, the acidification treatment affected the photosynthetic communities by changing the pigment proportion of the microbial mat, which probably resulted from a change in either their composition or their metabolisms (Figure 5). Concomitantly, an increase of dissolved oxygen concentration was observed in the water, likely due to an increase of the photosynthesis (Figure 5). The modifications of the conditions generated by the acidification impacted also the sulfur-oxidizers belonging to the *Sulfurimonas* genus and the Rhodobacteraceae family, which were significantly more abundant in this condition (Figure 4). In contrast, the warming of the water, characterized by an upsurge of salinity due to evaporation (Figure 5), increased the relative abundance of the sulphate-reducers *Desulfurimonas* (Figure 4). Finally, the warming and acidified treatment resulted in an increase of the water salinity but the dissolved oxygen concentration was not impacted (Figure 5), affecting the sulfur-oxidizers *Guyparkeria* (Figure 4).

Maintaining microbial mats in mesocosms following an experimental ecology approach allowed to apply conditions to simulate climate change, which is difficult to perform *in situ*. It also made possible to increase the number of replicates and analysis strengthening the statistical analyses. We were thus able to unveil the relationships between the microorganisms inhabiting the microbial mat and their environment, helpful information to refine the hypotheses of the impact of climate change at the ecosystem level. More generally, the gained information provides new insights to better understand the importance of microbial mats in their environment.

## 4. Conclusion and perspectives

Climate change is already happening, but it is difficult to determine its effect on complex microbial assemblages, particularly because *in situ* simulation experiments are difficult to perform. An alternative to overcome this difficulty is to follow an experimental ecology approach maintaining complex microbial community in mesocosm applying conditions simulating predicted climate change conditions. Microbial mats used allowed to unveil microbial structural modification in response to climate change. The mesocosm approach mimics *in situ* conditions excluding environmental complexity, which was useful to characterize the impact of each changed parameter independently. The total cost of the mesocosms set up was less than 3.000$. The described method in this paper allowed to simulate the change of two parameters, the water pH and temperature, alone and associated. The regular monitoring of several abiotic and biotic parameters and the presence of five replicates led to the complete description of the ecosystem. After applying climate change scenario, several physical-chemical and biological modifications were observed, which provide new insights on microbial mats behaviour. The gained information is useful to understand the impact of climate change at the ecosystem level. Microbial mats, easy to handle and to maintain in mesocosm, are thus precious complex microbial community model to investigate ecological issues of concern for coastal marine ecosystems. Mesocosms experiment can mimic as close as possible the environment but it can’t reproduce all *in situ* variations and stresses. The method presented could therefore be improved by adding rainfall cycles or seasonal variations. Additional analyses could be performed to specify the functional role of microbial mats in its environment, such as vertical microprofiling of oxygen and sulphur, or a day/night monitoring. This method could be adapted with additional climate change parameters as for example, deoxygenation or the alternance of drought and extreme rainfall. Other issues could also be investigated with this system, such as the impact of oil pollution, metals contamination, organic enrichment, etc. The presented method could also be extended on sediments coming from aquatic environments, as for example estuaries, beach, deep ocean, but also rivers, lakes, by adjusting the sampling method and the parameters applied.

## Data availability statement

The raw data supporting the conclusions of this article will be made available by the authors, without undue reservation.

## Author contributions

CM: formal analysis, methodology, and validation—original draft. RD, CD, and CC-L: funding acquisition, project administration, resources, and supervision. All authors: conceptualization, writing—review and editing, contributed to the article, and approved the submitted version.
